# Factors Associated with Catch-up Growth in Term, Asymmetrical Small-for-Gestational Age Infants in the First Year of Life

**DOI:** 10.5041/RMMJ.10452

**Published:** 2021-10-25

**Authors:** Sundar Sivakumar, Thasma Santhanakrishnan Arunprasath, Padmasani Venkat Ramanan

**Affiliations:** Department of Paediatrics, Sri Ramachandra Institute of Higher Education and Research, Chennai, Tamil Nadu, India

**Keywords:** Breastfeeding, diarrhea, intercurrent infections

## Abstract

**Introduction:**

Catch-up growth (CUG) in small-for-gestational age (SGA) infants is essential for their overall development. Knowledge about the factors influencing CUG might be critical in their effective management. Hence this study was performed with the aim of identifying factors that may influence CUG in SGA infants.

**Methods:**

Asymmetrical SGA infants born at term were included in the study as per defined criteria, and their demographic details were recorded. Anthropometric data, feeding practice details, and intercurrent illnesses data were collected on follow-up at 6 weeks, 6 months, and 12–15 months of age. Catch-up growth weight was defined as improvement of weight to the normal range of −2 to +2 weight-for-age *Z* score (WAZ). Analysis was carried out using SPSS Expand 17 software. Chi-square test was used to find association between variables. Logistic regression analysis was used to measure effect. A *P* value of less than 0.05 was taken as significant.

**Results:**

Out of 324 SGA infants born at term, 119 completed 12–15-month follow-up, of which 69.7% had achieved CUG weight. Exclusive breastfeeding >4 months, continued breastfeeding until 12–15 months, and absence of diarrheal episodes were positively associated with CUG. Pregnancy-induced hypertension, gestational diabetes, and maternal overweight/obesity were negatively associated with CUG. Maternal education status, conception age, gravida status, mode of delivery, vitamin D and iron supplementation, and intercurrent respiratory infections were not associated with CUG. On multivariate analysis, continued breastfeeding and absence of diarrheal episodes were independent factors associated with CUG.

**Conclusion:**

Breastfeeding practice, especially continued breastfeeding, and the absence of diarrheal illness are the key determinants for achieving CUG weight in term SGA infants, particularly in settings where resources are limited.

## INTRODUCTION

Catch-up growth (CUG) is the acceleration in growth that occurs postnatally in babies born small for gestational age (SGA). Weight catch-up is the decrease in the weight deficit between the individual and the reference mean for a healthy population that occurs over time. There are no international standards for definition of catch-up growth.^1^ One of the relative definitions of catch-up is improvement of weight to the normal range of −2 to +2 weight-for-age *Z* score (WAZ).^2^ Catch-up growth in SGA infants is associated with better neurobehavioral outcomes,^3^ prevention of metabolic consequences of early intrauterine growth retardation,^4^,^5^ prevention of persistent short stature,^6^ and better immune function.^7^ Correct understanding of the factors that positively and negatively influence CUG is vital for the appropriate management of SGA infants. The aim of this study was to follow up asymmetrical SGA babies born at term, once at 6–8 months, and again at 12–15 months of age, to identify factors associated with failure to achieve CUG.

## METHODS

This prospective cohort study was done with the approval of the Institutional Ethics Committee in the well-baby clinic of the Department of Paediatrics in a university teaching hospital in South India. The parents of SGA term infants born between August 2015 and July 2016 signed a written informed consent when their infants were 6–8 weeks of age, and indicated a willingness for the infants to undergo follow-up until 12–15 months of age. Infants were excluded if they had congenital malformations, length for gestational age less than the 10th centile at birth, chronic systemic illness, and neonatal intensive care unit hospitalization for more than 2 days. The mothers’ demographic and pregnancy details were obtained from their hospital records. Socioeconomic status was classified according to the Modified Kuppuswamy Scale.^8^ Birth anthropometry was noted from the discharge summary. The infants were followed up at 6 months and 12–15 months of age.

At each follow-up visit, the infants’ anthropometric measurements (weight, length, head circumference) were taken by a trained nurse in triplicate, and the average of the three readings was noted. Nude body weight was recorded using an electronic weighing scale to the nearest 5 g. Length was recorded using an infantometer to the nearest 0.1 cm, and the occipitofrontal circumference was measured using a non-stretchable tape measure. Anthropometry measurements were plotted on WHO growth charts. At 6 weeks, the infants were started on oral vitamin D drops (400 IU/day) and at 6 months on iron drops (elemental iron 1 mg/kg/day) and advised to continue until the final 12–15-month visit. At each subsequent visit, details regarding feeding, compliance to iron and vitamin D administration, and intercurrent illnesses were noted. The mothers’ height and weight were also measured at the final 12–15-month visit to assess the effect of maternal nutritional status in CUG of their infants.

Study definitions used were as detailed in Table 1.

**Table 1:** Study Definition Used for the Study.

Study Terminology	Definition
Catch-up growth (CUG) weight	WAZ score moving up to ≥ −2
Compliance to vitamin D and iron	Administration of vitamin drops at least 5 days/week as per predefined criteria
Diarrhea	Based on both clinical findings and official diagnosis
Exclusive breastfeeding (EBF)	World Health Organization (WHO) definition^9^
Infant anemia	Hemoglobin <11 g/dL
Maternal anemia	Hemoglobin <11 g/dL
Maternal obesity	BMI >30
Maternal overweight	BMI 25 to 29.9
Respiratory infection	Based on both clinical findings and official diagnosis
Small for gestational age (SGA)	Birth weight less than 10th percentile for gestation as per Intergrowth-21st charts^10^,^11^
Term	37 completed weeks as assessed by pregnancy dating scan

Data were analyzed using SPSS Expand 17 software. Chi-square test was used to find associations between variables. Variables which had significant association with achievement of CUG were noted, and the odds ratio (OR) was calculated. Those with a significant association were then analyzed with logistic regression model, and adjusted odds ratio was calculated to identify the independent variables. A *P* value of less than 0.05 was taken as significant.

## RESULTS

**Figure 1:**
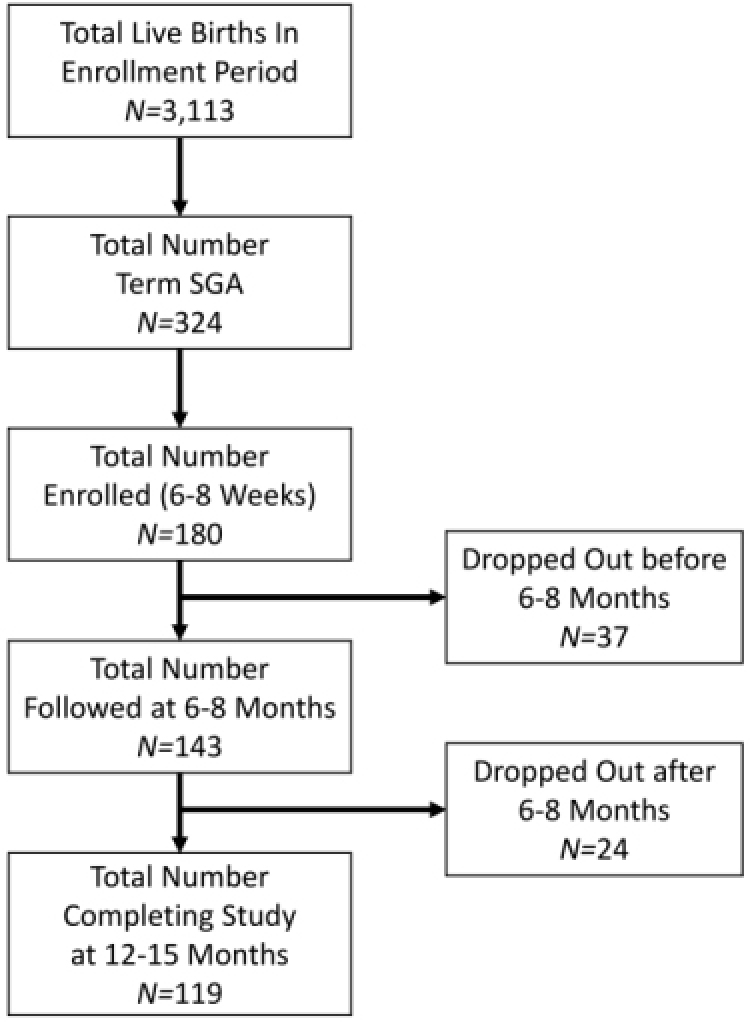
Study Group Flowchart.

There were 3,113 live births in the enrollment period, of which 324 were term SGA (10.4%). Of these, 180 SGA infants (104 boys, 76 girls) were enrolled at 6–8 weeks; 143 (86 boys, 57 girls) completed the 6–8-month follow-up, and 119 (62 boys, 57 girls) completed the 12–15-month follow-up (Figure 1). A WAZ score greater than −2 was achieved by 6 weeks in 41%, at 6 months in 79.8%, and by 12–15 months in 69.7% of the study group. Throughout the follow-up period, all children had a length and head circumference for age *Z* score of +2 to −2; at no time did the *Z* score exceed +2.

The clinico-sociodemographic factors associated with weight for age catch-up at 1 year were analyzed (Table 2).

**Table 2:** Univariate Analysis of Clinico-sociodemographic Factors Associated with Catch-up Growth at 1 Year.

Factors	Catch-up Growth Present (*n*=83)	Catch-up Growth Absent (*n*=36)	Total (*n*=119)	*P* Value
Mother education graduate	64 (77%)	31 (86.1%)	95 (79.8%)	0.261
Mother overweight/ obese	21 (25.3%)	18 (50%)	39 (32.8%)	0.008
Conception age <25	48 (57.8%)	18 (50%)	66 (55.5%)	0.430
Primipara	59 (71%)	21 (58.3%)	80 (67.2%)	0.173
Presence of antenatal medical condition	30 (36.1%)	24 (66.7%)	54 (45.4%)	0.002
PIH	10 (12%)	10 (27.8%)	20 (16.8%)	0.035
GDM	9 (10.8%)	9 (25%)	18 (15.1%)	0.048
Anemia	56 (67.5%)	23 (63.9%)	79 (66.4%)	0.704
Normal delivery	55 (66.3%)	19 (52.8%)	74 (62.2%)	0.163
Male infant	42 (50.6%)	20 (55.6%)	62 (52.1%)	0.619
Exclusive breastfeeding >4 months	42 (50.6%)	7 (19.4%)	49 (41.2%)	0.002
Continued breastfeeding until 12–15 months	72 (86.7%)	2 (5.6%)	74 (62.2%)	<0.0001
>1 Diarrhea episodes	53 (63.9%)	34 (94.4%)	87 (73.1%)	0.001
>2 Respiratory infection episodes	38 (45.8%)	23 (63.9%)	61 (51.3%)	0.07

Among the mothers, 55.5% were less than 25 years of age at the time of conception, 79.8% had a graduate education, 67.2% were primipara, and 62.2% had a normal vaginal delivery. There was no significant relationship between any of these factors and CUG in their infants. Overall, 32.8% mothers were either overweight or obese at infant age 12–15 months. Maternal overweight/obesity was significantly higher in the failed versus achieved CUG infants (50% versus 25.3%, respectively; *P* value 0.008).

The occurrence of pregnancy-induced hypertension (PIH) was higher among the failed versus achieved CUG infants (27.8% versus 12%, respectively; *P* value 0.035), as was gestational diabetes mellitus (GDM) (25% versus 10.8%, respectively; *P* value 0.048), and the difference was statistically significant.

Overall, exclusive breastfeeding (EBF) for less than 4 months was found in 70 infants (58.8%). Only 12 infants (overall 10.1%) were maintained on EBF for 6 months. The prevalence of EBF for >4 months was significantly greater in achieved versus failed CUG infants (50.6% versus 19.4%, respectively; *P* value 0.002). Breastfeeding together with complementary feeds was continued through to the 12–15-month follow-up visit by 74 mothers (62.2%). This practice of continued breastfeeding was significantly associated with CUG in the achieved versus failed infants (86.7% versus 5.6%; *P* value <0.0001, respectively). Although there was good compliance to vitamin D and iron supplementation in 33.6% and 36.9% of infants, respectively, there was no significant association between good compliance and CUG. Anemia was found in 66.4% of the children at the 12–15-month visit, with no significant association between anemia and CUG.

By 12–15 months of age, 73.1% of the children had experienced one or more episodes of diarrhea; the proportion of children with diarrhea was significantly lower in achieved versus failed CUG (63.9% versus 94.4%), respectively; *P* value 0.001). However, there was no significant difference in the proportion of infants with respiratory infection in either group (*P*=0.07) (Table 2).

On multivariate analysis of risk factors, continued breastfeeding until 12–15 months and absence of diarrheal episodes were found to be independent factors associated with CUG (Table 3). On further analysis it was found that 78.1% of children who had continued breastfeeding had no episode of diarrhea in the first year as compared to 21.9% who had been breastfed for less than 12–15 months (*P* value 0.03).

**Table 3:** Univariate and Multivariate Analyses of Factors Associated with Catch-up Growth at 1 Year.

Factors	Unadjusted Odds Ratio	*P* Value	Adjusted Odds Ratio	*P* Value
Maternal overweight/obesity	0.34 (0.15–0.77)	0.08	1.463 (0.327–6.558)	0.619
Absence of maternal antenatal risk factors	3.53 (1.55–8.06)	0.002	3.328 (0.315–35.166)	0.318
PIH	0.36 (0.13–0.95)	0.035	0.699 (0.312–8.114)	0.792
GDM	0.36 (0.13–1.02)	0.048	0.879 (0.053–14.475)	0.928
Duration of EBF >4 months	4.24 (1.67–10.76)	0.02	1.590 (0.312–8.114)	0.577
Continued breastfeeding till 12–15 months	111.27 (23.6–529.9)	<0.0001	120.47 (19.73–735.48)	<0.0001
Diarrheal episodes <1	9.62 (2.16–42.8)	0.001	8.93 (1.18–67.24)	0.034

EBF, exclusive breastfeeding; GDM, gestational diabetes mellitus; PIH, pregnancy-induced hypertension.

## DISCUSSION

In the present study, 69.7% of SGA infants achieved CUG in weight at 12–15 months of age. We also studied the maternal–infant factors associated with weight CUG. Absence of antenatal risk factors, EBF for 4 months, continued breastfeeding until 12–15 months, and fewer diarrheal episodes in the first 12–15 months were favorably associated with CUG. However, on multivariate analysis, continued breastfeeding until 12–15 months and absence of diarrheal episodes were the only two independent factors associated with CUG.

There was no significant association between CUG and other maternal factors (mother’s age at conception, education, gravida status, and mode of delivery) or infant factors such as gender, mode of delivery, or number of respiratory infections during infancy.

Maternal PIH or GDM were more prevalent in the failed CUG infants. Previous studies have shown that CUG is not influenced by antenatal factors.^12^,^13^ The association found in our study may be attributed to the severity of antenatal medical conditions and other confounding factors such as maternal age, pregnancy weight gain, or parity, although no significance was noted via multivariate analysis.

Similar observations of breastfeeding being a key factor influencing CUG, independently of confounding factors, have been made in previous studies.^14^–^16^ This breastfeeding practice also influences the incidence of intercurrent infections, particularly diarrheal episodes. It is well known that diarrheal episodes have a negative impact on the nutritional status of infants.^17^

The overall EBF rate until 6 months in our study group was 10.1% as compared to the national average of 50%.^18^ This may be due to maternal anxiety regarding low birth weight and postnatal weight gain, which prompted early introduction of complementary feeds. However, this is purely conjectural as we did not probe into the reasons for early initiation of complementary feeds in our study.

Vitamin D and iron supplementation did not have an impact on CUG. It is well established that these are type 1 nutrients which, when deficient, cause characteristic clinical symptoms associated with the dysfunction of a particular biochemical pathway, and are unrelated to growth failure.^19^

Growth restriction in SGA can occur in the first or later trimesters. Restricted growth in the later trimester is mainly due to maternal factors and an unfavorable intrauterine environment. This pattern of growth restriction results in asymmetrical intrauterine growth restriction. After delivery these babies are expected to achieve CUG. However, multiple external factors may influence it.

The demographic profile and socioeconomic and cultural factors for all study participants who completed final follow-up were similar and did not change during follow-up.

A major limitation of the study was the attrition rate and failure to follow up all recruited infants, which can be considered as attrition bias. However, we assume that this bias might not have affected the study results as attrition was for social reasons and not intentional. The primary reason for follow-up failure was migration of the mother–infant duo owing to the local cultural practice of having the delivery in the mother’s place of pre-marriage residence, and moving back to her husband’s residence after 3–5 months. Another limitation is that this observation was made in a developing country, with most of the families belonging to the lower middle socioeconomic class, and the results cannot be generalized for developed countries.

## CONCLUSION

Overall, 69.7% of term SGA babies showed CUG according to the WAZ score at 12–15 months. The independent factors associated with CUG were continued breastfeeding until 12–15 months and the absence of diarrheal episodes during the study period. These findings are from a resource-limited setting and cannot be generalized for all countries.

## Abbreviations

BMI: body mass index
CUG: catch-up growth
EBF: exclusive breastfeeding
GDM: gestational diabetes mellitus
PIH: pregnancy-induced hypertension
SGA: small for gestational age
WAZ: weight-for-age *Z* score
WHO: World Health Organization

## References

[1] Martin A, Connelly A, Bland RM, Reilly JJ (2017). Health impact of catch-up growth in low-birth weight infants: systematic review, evidence appraisal, and meta-analysis. Matern Child Nutr.

[2] Tenhola S, Martikainen A, Rahiala E, Herrgârd E, Halonen P, Voutilainen R (2000). Serum lipid concentrations and growth characteristics in 12-year-old children born small for gestational age. Pediatr Res.

[3] Takeuchi A, Yorifuji T, Nakamura K (2018). Catch-up growth and neurobehavioral development among full-term, small-for-gestational-age children: a nationwide Japanese population-based study. J Pediatr.

[4] Saenger P, Czernichow P, Hughes I, Reiter EO (2007). Small for gestational age: short stature and beyond. Endocr Rev.

[5] Toumba M, Hadjidemetriou A, Topouzi M (2005). Evaluation of the auxological and metabolic status in prepubertal children born small for gestational age. J Pediatr Endocrinol Metab.

[6] Karlberg J, Albertsson-Wikland K (1995). Growth in full-term small-for-gestational-age infants: from birth to final height. Pediatr Res.

[7] Xiong F, Huo TZ, Li P, Yang F, Mao M (2015). Sichuan Da Xue Xue Bao Yi Xue Ban.

[8] Khairnar MR, Wadgave U, Shimpi PV (2017). Kuppuswamy’s socio-economic status scale: a revision of occupation and income criteria for 2016. Indian J Pediatr.

[9] World Health Organization (2003). Global Strategy for Infant and Young Child Feeding.

[10] University of Oxford and Intergrowth-21st International Standards for Size at Birth (Boys). Updated March 2015. The Global Health Network Website.

[11] University of Oxford and Intergrowth-21st International Standards for Size at Birth (Girls).

[12] Huh J, Kwon JY, Kim HR (2018). Comparison of postnatal catch-up growth according to definitions of small for gestational age infants. Korean J Pediatr.

[13] Khadilkar VV, Mandlik RM, Palande SA (2016). Growth status of small for gestational age Indian children from two socioeconomic strata. Indian J Endocrinol Metab.

[14] Lucas A, Fewtrell MS, Davies PS, Bishop NJ, Clough H, Cole TJ (1997). Breastfeeding and catch-up growth in infants born small for gestational age. Acta Paediatr.

[15] Tudehope D, Vento M, Bhutta Z, Pachi P (2013). Nutritional requirements and feeding recommendations for small for gestational age infants. J Pediatr.

[16] Çamurdan MO, Çamurdan AD, Polat S, Beyazova U (2011). Growth patterns of large, small, and appropriate for gestational age infants: impacts of long-term breastfeeding: a retrospective cohort study. J Pediatr Endocrinol Metab.

[17] Richard SA, Black RE, Gilman RH (2014). Catch-up growth occurs after diarrhea in early childhood. J Nutr.

[18] (2018). International Institute for Population Sciences (IIPS), ICF. National Family Health Survey (NFHS-4), 2015.

[19] Shoham J, Duffield A (2009). Proceedings of the World Health Organization/UNICEF/World Food Programme/United Nations High Commissioner for Refugees Consultation on the management of moderate malnutrition in children under 5 years of age. Food Nutr Bull.

